# Unmasking a High-Grade Malignant Neoplasm: A Rare Case of Small Bowel Melanoma

**DOI:** 10.7759/cureus.107132

**Published:** 2026-04-15

**Authors:** Lakshmisree A Vemulakonda, Deepti Reddi, Yongjun Liu

**Affiliations:** 1 Department of Laboratory Medicine and Pathology, University of Washington Medical Center, Seattle, USA; 2 Department of Laboratory Medicine and Pathology, University of Washington, Seattle, USA

**Keywords:** braf v600 mutation, malignant melanoma, pathology, peritheliomatous growth pattern, small bowel

## Abstract

Malignant melanoma of the small bowel is a rare form of gastrointestinal malignancy. This case report describes a 70-year-old woman who presented with symptomatic iron deficiency anemia, progressive abdominal pain, weight loss, and other nonspecific symptoms, prompting an extensive diagnostic workup. Initial endoscopic evaluations were unremarkable, leading to further investigations, including capsule endoscopy and radiologic studies, which identified a jejunal mass. Histopathological and immunohistochemical analyses confirmed the diagnosis of malignant melanoma, and molecular testing identified a BRAF V600 mutation. The patient subsequently underwent surgical resection. This case highlights the diagnostic challenges of small bowel melanoma and the importance of comprehensive clinical, histopathological, and molecular evaluation in establishing the diagnosis. Given the aggressive nature of this malignancy, early diagnosis and timely intervention are essential for improving patient outcomes.

## Introduction

Malignant melanoma of the small bowel is an extremely rare malignancy, comprising 1-3% of gastrointestinal (GI) cancers [1-4]. While the small intestine is a common site for metastatic melanoma in the GI tract, primary small bowel melanomas are exceedingly uncommon, with only a limited number of cases documented in the literature [1-4]. The pathogenesis remains controversial, with proposed origins including melanocytes within the alimentary tract [5], neural crest-derived amine precursor uptake and decarboxylation (APUD) cells [6], or Schwann cells [7].

Clinically, gastrointestinal melanoma often presents with nonspecific symptoms, including abdominal pain, anemia, gastrointestinal bleeding, weight loss, and fatigue, which frequently lead to delayed diagnosis [2]. In many cases, patients remain asymptomatic until advanced disease or complications such as obstruction or bleeding occur. The clinical course is typically aggressive, with a high propensity for early metastasis. Prognosis is generally poor, with a reported median survival of only a few months and low long-term survival rates [2].

This report describes a case of a high-grade malignant neoplasm of the small bowel referred to our institution for further evaluation that was ultimately diagnosed as small bowel melanoma. The case highlights the importance of advanced imaging, comprehensive histopathological evaluation, and molecular analysis in establishing an accurate diagnosis and guiding treatment decisions.

## Case presentation

A 70-year-old woman presented with progressive abdominal pain, night sweats, weight loss, decreased appetite, lightheadedness, and intermittent nausea, vomiting, and diarrhea. Laboratory evaluation demonstrated iron deficiency anemia and stool guaiac positivity (Table 1), raising concern for gastrointestinal blood loss. Other laboratory parameters, including renal and liver function tests, were largely within normal limits (Table 1).

**Table 1:** Summary of laboratory results RBC: red blood cell, MCV: mean corpuscular volume, MCH: mean corpuscular hemoglobin, MCHC: mean corpuscular hemoglobin concentration, RDW: red cell distribution width, WBC: white blood cell, Hb: hemoglobin, RET-He: reticulocyte hemoglobin equivalent, TIBC: total iron-binding capacity, CO₂: carbon dioxide, BUN: blood urea nitrogen, eGFR: estimated glomerular filtration rate, AST: aspartate aminotransferase, ALT: alanine aminotransferase.

Category	Test	Result	Reference Range	Interpretation
Hematology				
	Hemoglobin	7.1–8.7 g/dL	11.5-15.0 g/dL	↓ Anemia
	Hematocrit	23.4-28.7%	34.8-45.0%	↓
	RBC count	3.31-3.45 M/µL	3.75-5.07 M/µL	↓
	MCV	82-83 fL	80-100 fL	Normocytic (borderline microcytic)
	MCH	~25 pg	25.9-34.2 pg	↓
	MCHC	~30-31 g/dL	31.5-36.5 g/dL	↓
	RDW	15.3-15.7%	11.5-14.2%	↑
	Platelets	331-332 K/µL	150-400 K/µL	Normal
	WBC	9.1-10.0 K/µL	4.0-11.0 K/µL	Normal
Reticulocyte studies				
	Reticulocyte %	2.3%	0.4-2.5%	Normal / slightly ↑
	Immature reticulocyte fraction	28.5%	1.9-15.1%	↑
	Reticulocyte Hb (RET-He)	20.5 pg	30-37 pg	↓ (iron deficiency)
Iron studies				
	Ferritin	8.2 ng/mL	7-271 ng/mL	↓ (iron deficiency)
	Iron	151 µg/dL	28-170 µg/dL	Hemolyzed specimen
	TIBC	345 µg/dL	285-513 µg/dL	Normal
	Transferrin	245 mg/dL	202-364 mg/dL	Normal
	% Transferrin saturation	44%	15-50%	Normal
Chemistry panel				
	Sodium	141 mmol/L	136-145 mmol/L	Normal
	Potassium	4.2 mmol/L	3.5-5.1 mmol/L	Normal
	Chloride	112 mmol/L	98-111 mmol/L	↑
	CO₂	25 mmol/L	21-32 mmol/L	Normal
	Glucose	110 mg/dL	70-99 mg/dL	Mildly ↑
	BUN	9 mg/dL	9-23 mg/dL	Normal
	Creatinine	0.76 mg/dL	0.55-1.02 mg/dL	Normal
	eGFR	85 mL/min/1.73 m²	≥60	Normal
	Calcium	8.7 mg/dL	8.7-10.4 mg/dL	Normal
Liver function tests				
	AST	13 U/L	~10-40 U/L	Normal
	ALT	22 U/L	~7-56 U/L	Normal
	Alkaline phosphatase	114 U/L	~44-147 U/L	Normal
	Total bilirubin	0.6 mg/dL	~0.2-1.2 mg/dL	Normal
	Albumin	3.4 g/dL	3.5-5.0 g/dL	Slightly ↓
Other				
	Vitamin B12	473 pg/mL	211-911 pg/mL	Normal

An extensive diagnostic workup was subsequently undertaken, and the clinical timeline of the disease course is summarized in the Appendices. 

**Figure 1:**
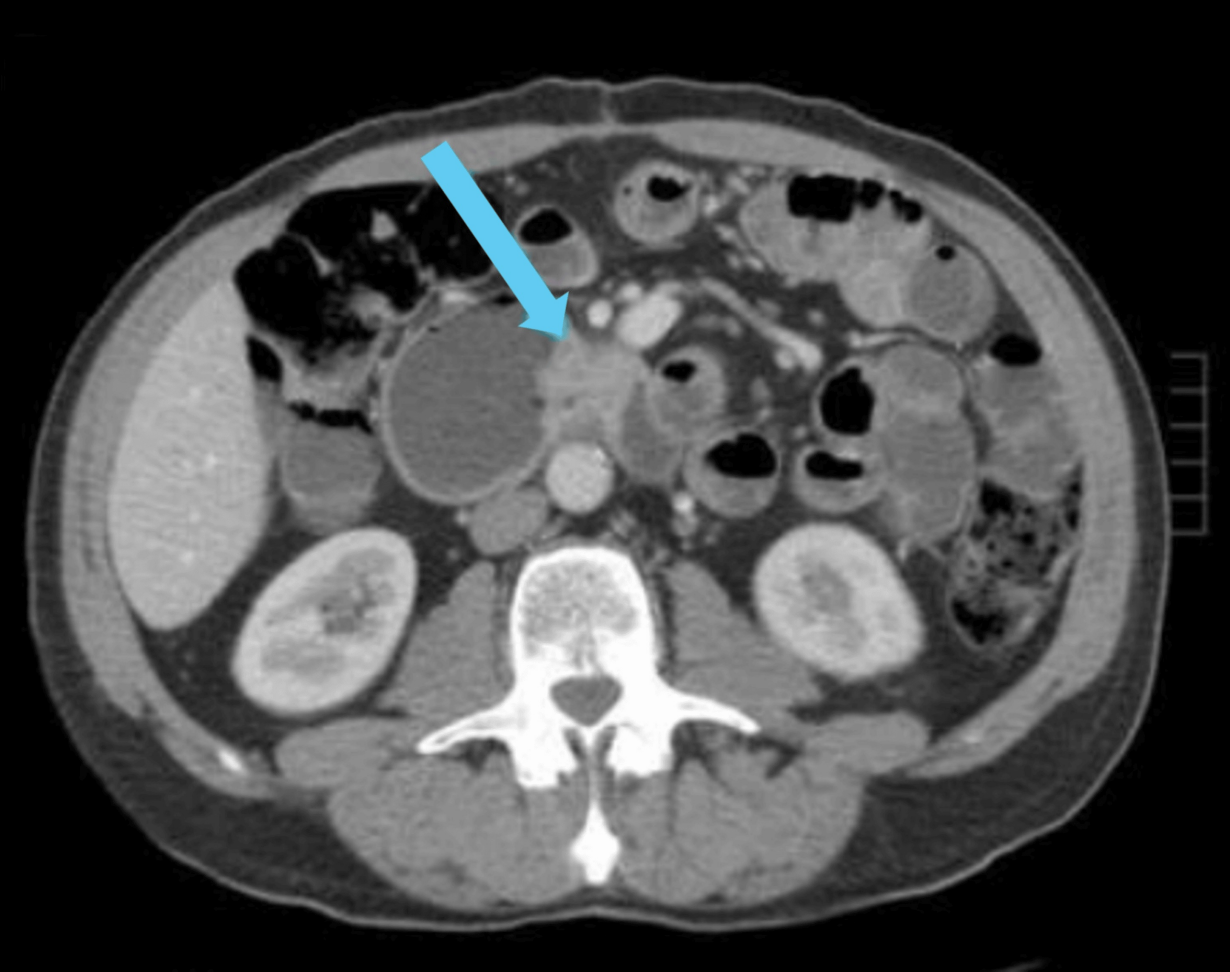
CT scan showing enlarging masses (arrow) in the left upper abdomen, suggesting small bowel lymphoma with adenopathy

Initial investigations, including colonoscopy and esophagogastroduodenoscopy, were unremarkable, revealing only non-thrombosed internal hemorrhoids and diverticulosis. Five months later, wireless capsule endoscopy identified a large jejunal ulcer involving at least two-thirds of the bowel circumference. Computed tomography (CT) imaging demonstrated irregular bowel wall thickening, aneurysmal dilation of a jejunal segment, and enlarged mesenteric lymph nodes, raising suspicion for lymphoma. A follow-up CT scan confirmed enlarging masses in the left upper abdomen, suggesting small bowel lymphoma with adenopathy (Figure 1).

A biopsy of the jejunal lesion was performed at an outside institution for flow cytometry and histologic evaluation. The flow cytometry sample was paucicellular, and no abnormal B-cell or T-cell populations were identified. Histologic examination showed a poorly differentiated malignant neoplasm. Tumor cells were focally positive for HMB45 and negative for pancytokeratin, synaptophysin, chromogranin, CD45, CD20, and CD3. Based on these findings, the outside diagnosis was rendered as “poorly differentiated malignant neoplasm.”

**Figure 2:**
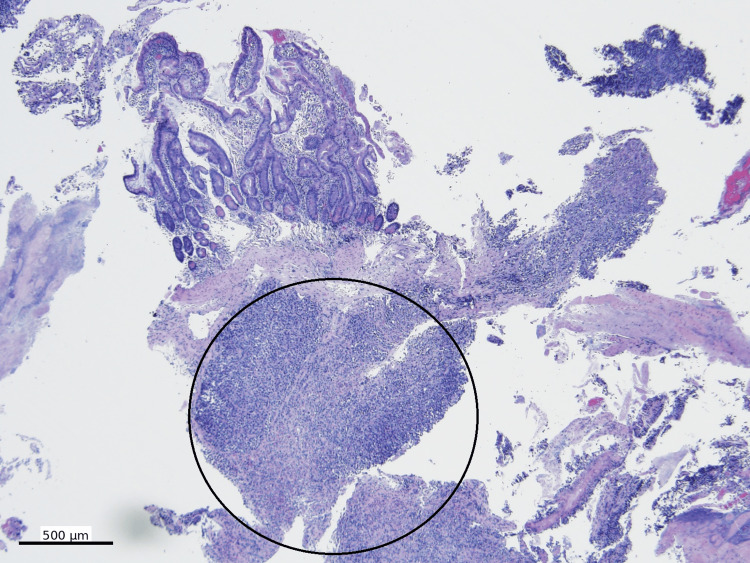
Low-power view of the mass Sheets of discohesive malignant neoplastic cells (open circle) with marked cytologic atypia and extensive necrosis (H&E, 40x)

The biopsy case was further referred to our institution for consultation. Hematoxylin and eosin (H&E)-stained sections showed sheets of discohesive malignant neoplastic cells with marked cytologic atypia and extensive necrosis (Figure 2).

**Figure 3:**
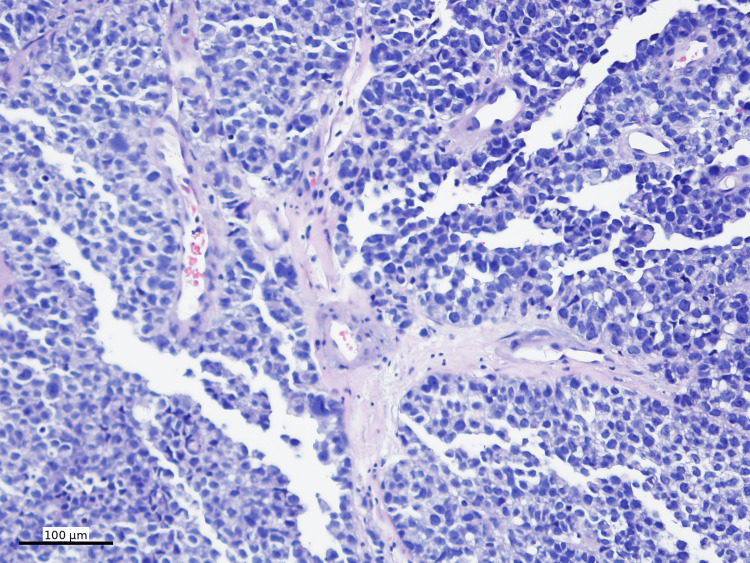
High-power view of the mass Focal pseudopapillary structures with a peritheliomatous growth pattern (H&E, 200x)

Focal pseudopapillary structures with a peritheliomatous growth pattern were present (Figure 3).

**Figure 4:**
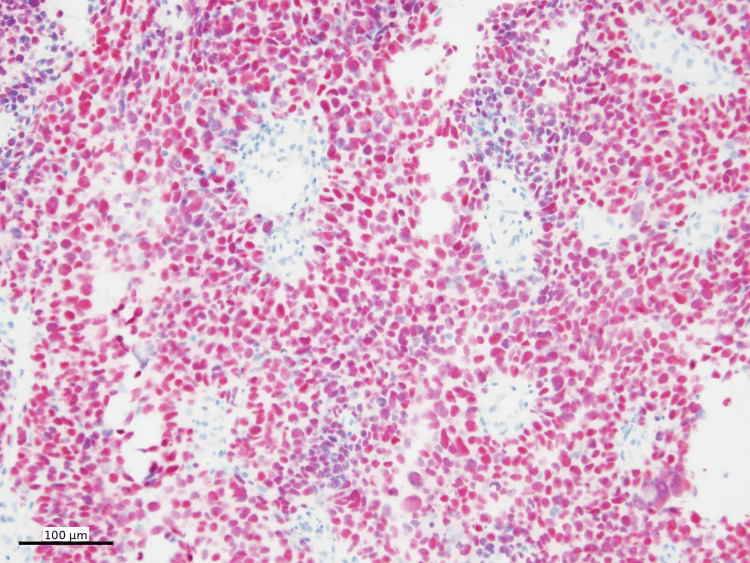
SOX10 immunohistochemistry SOX10 IHC (nuclear, Fast Red) shows diffuse positivity in tumor cells (100x)

**Figure 5:**
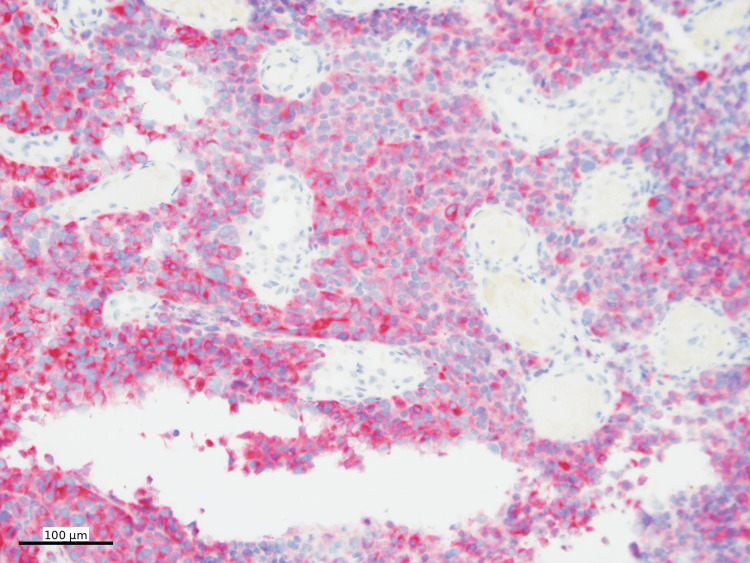
Melan-A immunohistochemistry Melan-A IHC (cytoplasmic, Fast Red) shows diffuse positivity in tumor cells (100x)

**Figure 6:**
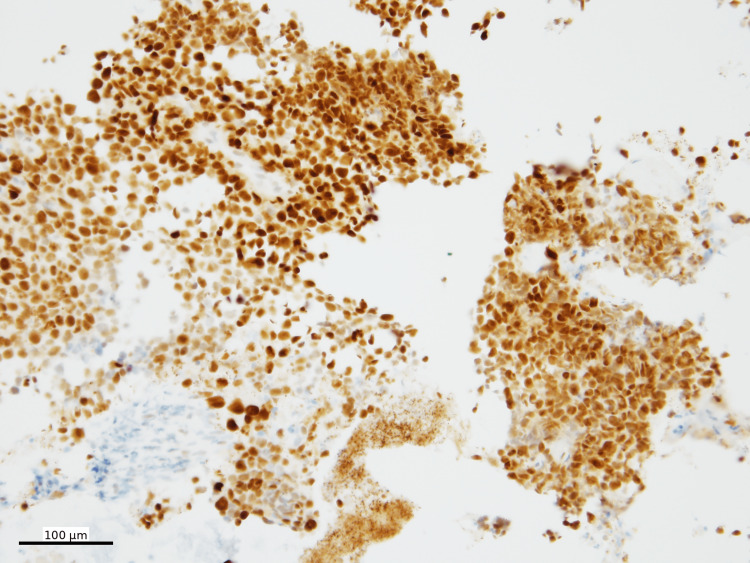
PRAME immunohistochemistry PRAME IHC (nuclear, DAB) shows diffuse positivity in tumor cells (100x)

**Figure 7:**
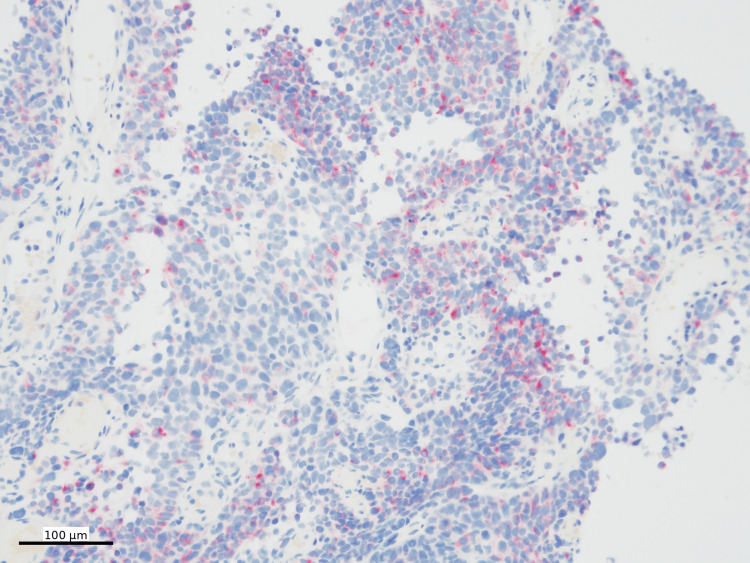
HMB45 immunohistochemistry HMB45 IHC (cytoplasmic, Fast Red) shows patchy positivity in tumor cells (100x)

The histologic findings raised a broad differential diagnosis, including lymphoma, malignant melanoma, malignant gastrointestinal neuroectodermal tumor (GNET), and clear cell sarcoma, among others. Additional ancillary studies were performed. Immunohistochemical staining was performed using standard chromogens, with SOX10, Melan-A, and HMB45 visualized using Fast Red chromogen and PRAME using DAB. Immunohistochemical stains showed that the neoplastic cells were diffusely positive for SOX10 (Figure 4), Melan-A (Figure 5), and PRAME (Figure 6), with patchy positivity for HMB45 (Figure 7).

**Figure 8:**
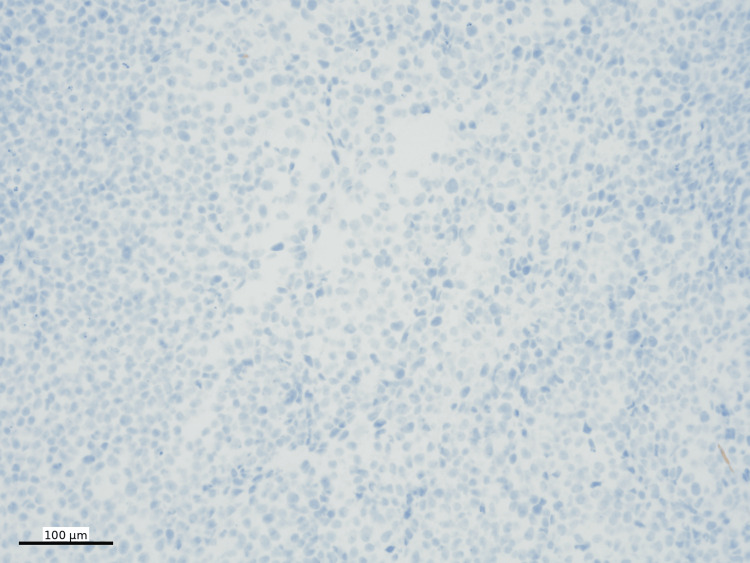
Pancytokeratin immunohistochemistry Pancytokeratin IHC (nuclear, DAB) is negative in tumor cells (100x)

**Figure 9:**
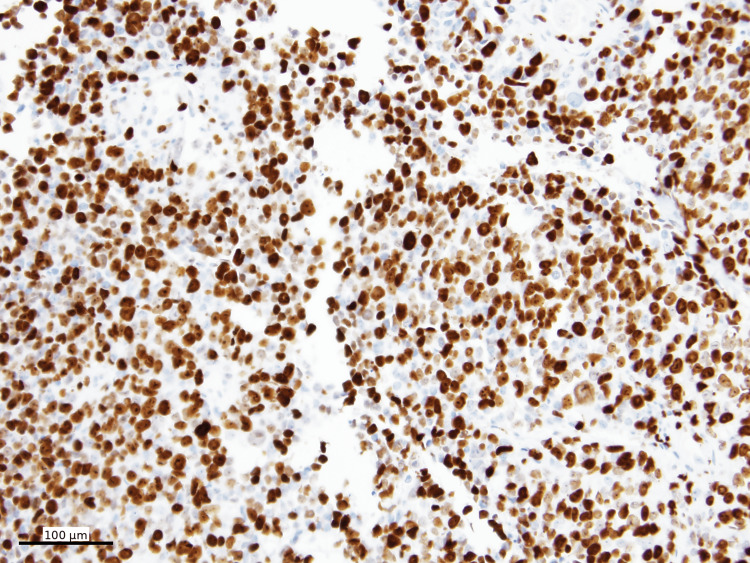
Ki-67 Ki-67 proliferative index (nuclear, DAB) is greater than 90% (100x)

The tumor was negative for a panel of lymphoma markers, as well as other markers including pancytokeratin (Figure 8), E-cadherin, desmin, SMA (smooth muscle actin), myogenin, ALK1, EMA, p40, S100, and ERG (erythroblast transformation-specific-related gene). The Ki-67 proliferation index was greater than 90% (Figure 9).

Mismatch repair (MMR) protein analysis showed loss of nuclear expression of MSH6, with retained expression of MLH1, MSH2, and PMS2. Taken together, the findings were most consistent with malignant melanoma. Notably, no pigments were present in the malignant cells, consistent with an amelanotic presentation. Additional molecular testing identified a BRAF V600K mutation (1798_1799delGTinsAA), further supporting the diagnosis. KRAS and NRAS mutations were negative by PCR analysis. 

**Figure 10:**
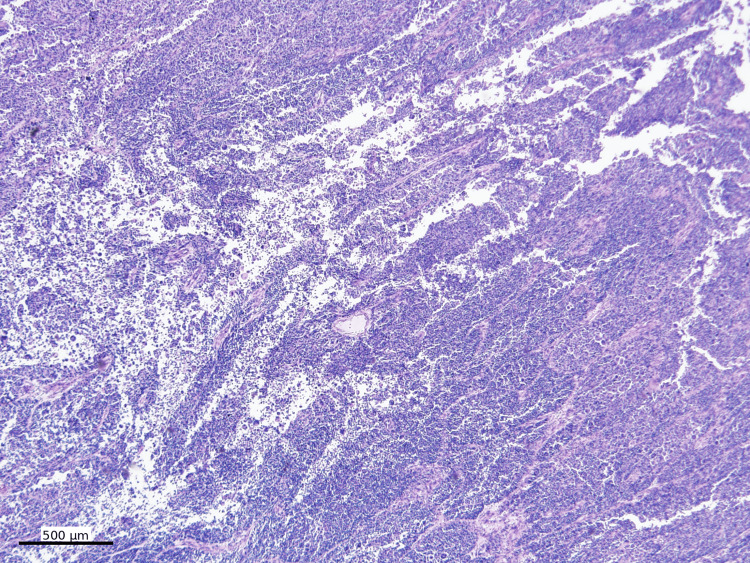
Hematoxylin and eosin stain of resection Resection of the tumor shows sheets of high-grade epithelioid neoplastic cells with focal peritheliomatous growth (H&E, 40x)

Subsequent small bowel resection revealed a 12 cm lesion. Histologic examination showed sheets of high-grade epithelioid neoplastic cells with focal peritheliomatous growth, morphologically similar to the biopsy specimen (Figure 10).

**Figure 11:**
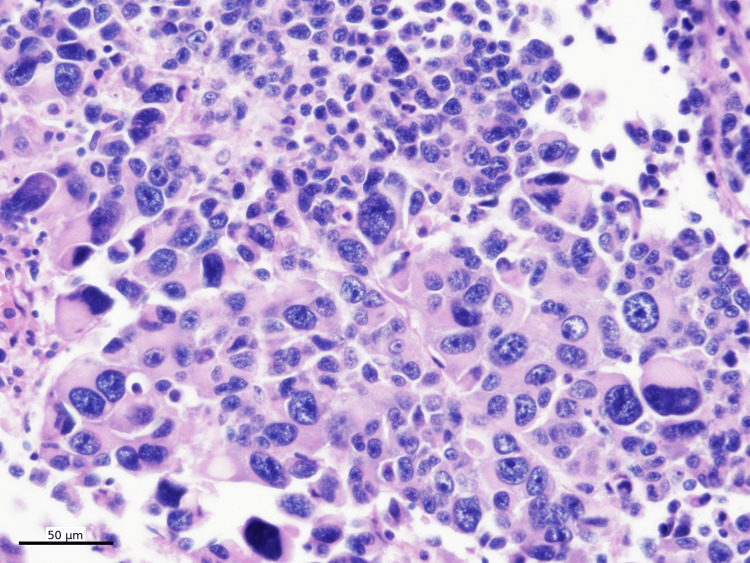
Hematoxylin and eosin stain with rhabdoid feature Focal rhabdoid-like features and occasional giant tumor cells were identified (H&E, 400x)

In addition, focal rhabdoid-like features and occasional giant tumor cells were identified (Figure 11).

**Figure 12:**
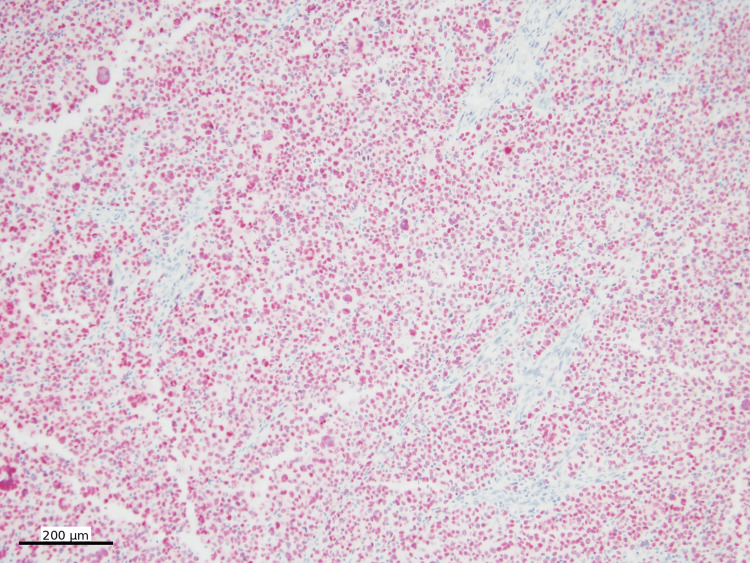
Resection, SOX10 immunohistochemistry SOX10 IHC (nuclear, Fast Red) is diffusely positive in tumor cells in resection specimen (100x)

**Figure 13:**
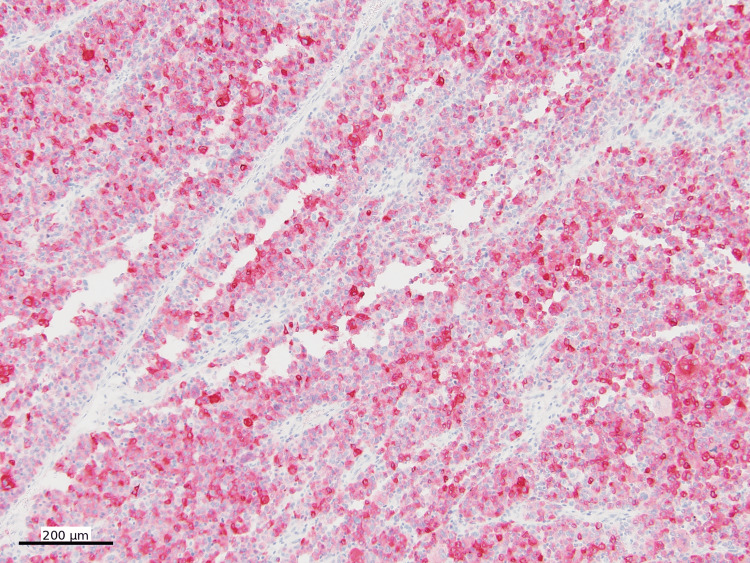
Resection, Melan-A immunohistochemistry Melan-A IHC (cytoplasmic, Fast Red) is diffusely postiive in tumor cells in resection specimen (100x)

The immunoprofile was identical to that of the biopsy specimen, with diffuse positivity for SOX10 (Figure 12) and Melan-A (Figure 13), supporting the diagnosis of malignant melanoma. The tumor infiltrated through the muscularis propria into the subserosa, without evidence of macroscopic perforation. Three of 11 lymph nodes were positive for metastasis. A thorough clinical investigation failed to identify another primary site of melanoma, supporting the diagnosis of primary small bowel melanoma. The tumor was staged as pT3N1bM0. 

## Discussion

In the GI tract, the jejunum and ileum are the most common sites for metastatic melanoma, likely due to the expression of CCR9 on melanoma cells, which binds to CCL25, highly expressed in the small intestine [8]. Primary small bowel melanoma is exceedingly rare, with only a few cases reported in the literature [9-11]. There is ongoing debate as to whether these are true primary tumors or metastases from an unknown site. Diagnostic criteria for true primary small bowel melanoma have been proposed. Blecker et al. [3] suggested that diagnosis requires a solitary tumor confined to the mucosa with associated intramucosal melanocytic lesions and no evidence of cutaneous or mucosal melanoma. Sacks et al. [12] proposed additional criteria, including the absence of distant metastases beyond regional lymph nodes and relapse-free survival of at least one year after diagnosis.

Primary small bowel melanomas may demonstrate diverse histologic features and often resemble those from other anatomical sites. The tumors typically consist of a mixture of epithelioid and spindled cells. The epithelioid component is characterized by large, pleomorphic cells with abundant cytoplasm and prominent eosinophilic nucleoli. Tumor cells often show poor cohesion, particularly in epithelioid regions, and may contain melanin pigment or may be completely amelanotic. A peritheliomatous growth pattern, characterized by a rim of viable tumor cells encircling a central blood vessel, has been described as a distinctive cytologic feature of metastatic melanoma in fine-needle aspiration (FNA) samples [13]. This pattern arises due to the inherently discohesive nature of melanoma cells, which tend to cluster around vascular structures rather than forming cohesive sheets following aspiration. The presence of this pattern in the current case suggests that it may not be exclusive to cytologic preparations and could also be observed in histologic sections of primary GI mucosal melanoma.

The differential diagnosis can be broad, depending on the tumor's histomorphology, and may include poorly differentiated carcinoma, neuroendocrine neoplasm, malignant peripheral nerve sheath tumor, gastrointestinal stromal tumor, sarcoma, and lymphoma. Immunohistochemistry is essential for establishing an accurate diagnosis. Although not routinely used in clinical practice, electron microscopy can aid in confirmation by identifying melanosomes and premelanosomes within tumor cells. Molecular genetic testing is also valuable, both in detecting underlying mutations and in guiding targeted therapy.

Small bowel melanomas tend to infiltrate laterally through the submucosa and can penetrate to varying depths within the bowel wall. Even small lesions are almost always associated with metastasis to the submucosal layer, where they often appear grossly and microscopically well circumscribed. As they progress, these tumors may ulcerate the overlying mucosa and invade deeper layers of the bowel wall [3].

From a genetic standpoint, GI mucosal melanomas often have different molecular profiles compared to cutaneous melanomas. BRAF mutations, which are common in cutaneous melanomas, are relatively less prevalent in GI mucosal melanomas [14]. In contrast, mutations in the KIT and SF3B1 genes are more frequently observed in the latter [15]. Although additional data are needed, given the rarity of this entity, the findings suggest potentially distinct molecular pathways involved in the pathogenesis of GI mucosal melanomas.

Clinically, small bowel melanomas often present diagnostic challenges due to their nonspecific symptoms. Many patients remain asymptomatic until severe complications, such as intestinal obstruction or bleeding, develop. Because these tumors are often difficult to detect with conventional endoscopy, alternative imaging techniques, such as CT and PET scans, play an important role in diagnosis. The prognosis is generally poor due to its aggressive nature and rich vascular and lymphatic supply. Median survival is approximately 4-6 months, with a five-year survival rate of less than 10% [16]. There is currently no standardized treatment protocol; however, surgical resection remains the cornerstone of therapy, often combined with immunotherapy. Chemoradiotherapy has shown limited benefit in improving overall survival.

## Conclusions

Primary small bowel melanoma is an extremely rare and aggressive malignancy that poses significant diagnostic challenges due to its nonspecific clinical presentation and overlapping histologic features with other tumors. This case highlights the importance of integrating clinical history, imaging findings, histopathology, immunohistochemistry, and molecular studies to achieve an accurate diagnosis. Increased awareness of this entity may facilitate earlier recognition and appropriate management. Distinguishing primary from metastatic melanoma requires careful clinical correlation, exclusion of other primary sites, and consideration of proposed diagnostic criteria, including solitary lesion, absence of prior melanoma, and limited metastatic disease. Sharing such cases contributes to improved diagnostic accuracy and ultimately better patient care.

## Figures and Tables

**Figure 14:**
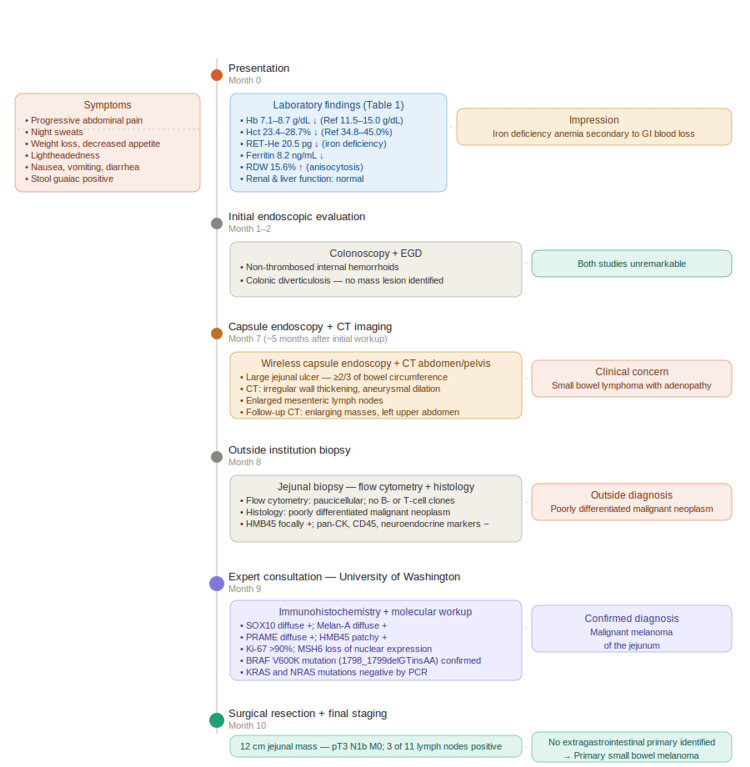
Clinical timeline This figure demonstrates the clinical timeline of disease course
